# The draft genome sequence of an upland wild rice species, *Oryza granulata*

**DOI:** 10.1038/s41597-020-0470-2

**Published:** 2020-04-29

**Authors:** Cong Shi, Wei Li, Qun-Jie Zhang, Yun Zhang, Yan Tong, Kui Li, Yun-Long Liu, Li-Zhi Gao

**Affiliations:** 1 Plant Germplasm and Genomics Center, Germplasm Bank of Wild Species in Southwestern China, Kunming Institute of Botany, Chinese Academy of Sciences, Kunming, 650204 China; 2 University of Chinese Academy of Sciences, Beijing, 100039 China; 3 Institution of Genomics and Bioinformatics, South China Agricultural University, Guangzhou, 510642 China

**Keywords:** Genome evolution, Plant evolution

## Abstract

Exploiting novel gene sources from wild relatives has proven to be an efficient approach to advance crop genetic breeding efforts. *Oryza granulata*, with the GG genome type, occupies the basal position of the *Oryza* phylogeny and has the second largest genome (~882 Mb). As an upland wild rice species, it possesses renowned traits that distinguish it from other *Oryza* species, such as tolerance to shade and drought, immunity to bacterial blight and resistance to the brown planthopper. Here, we generated a 736.66-Mb genome assembly of *O. granulata* with 40,131 predicted protein-coding genes. With Hi-C data, for the first time, we anchored ~98.2% of the genome assembly to the twelve pseudo-chromosomes. This chromosome-length genome assembly of *O. granulata* will provide novel insights into rice genome evolution, enhance our efforts to search for new genes for future rice breeding programmes and facilitate the conservation of germplasm of this endangered wild rice species.

## Abstract (summary)

**Table:** 

Measurement(s)	DNA • RNA • transcriptome • genome coverage • sequence_assembly • sequence feature annotation
Technology Type(s)	DNA sequencing • RNA sequencing • flow cytometry method • computational modeling technique • sequence assembly process • sequence annotation
Sample Characteristic - Organism	Oryza granulata

Machine-accessible metadata file describing the reported data: 10.6084/m9.figshare.12063198

## Background & Summary

As one of the most important crops in the world, rice is the most water-consuming cereal. Rice cultivation and yield depend greatly on water resources. The genetic breeding of drought-tolerant rice is a promising direction under the currently mounting water shortage. However, most species in the genus *Oryza* prefer moist and even aquatic habitats, and thus, upland rice breeding is very demanding due to the scarcity of gene sources with drought tolerance in the genus *Oryza*.

The genus *Oryza* contains more than twenty species, including two cultivated species domesticated independently from different wild species^1^. Compared to the majority of other grass species, *Oryza* species have relatively small genomes and abundant morphological and ecological diversity. *Oryza granulata* occupies the basal position of the *Oryza* phylogeny, first diverging from other members of the genus 8.8–10.2 million years ago^2^. *O. exasperata* (A. Braun) Heer was identified according to a spikelet fossil, which was found in an excavation site of Miocene age in Germany and appears to resemble the spikelet of extant *O. granulata* based on its morphology^3,4^. As an upland wild rice species, *O. granulata* possesses renowned traits that distinguish it from other *Oryza* species, such as tolerance to shade and drought, immunity to bacterial blight and resistance to the brown planthopper^5–7^. Because of the distant evolutionary relationship of this species with cultivated rice, it has long been challenging to apply conventional methods used in rice breeding programmes to it.

Compared to that for other wild species closely related to cultivated rice, little effort has been made to perform genetic studies and germplasm exploitation in *O. granulata* due to the lack of a high-quality genome assembly. Among the diploid *Oryza* species, *O. granulata* (GG genome type) has the second largest genome (~882 Mb), smaller than only that of *O. australiensis* (~965 Mb, EE genome type)^8^, which is approximately two times larger than the rice genome (~420 Mb, AA genome type)^9^. The two-fold increase in genome size is mainly due to the accumulation of transposable elements (TEs) in *O. granulata*, which may have seriously eroded genome collinearity compared with that in other related rice species^8,10,11^. In the last decade, great progress has been made in comparative genomics of cultivated rice and its wild relatives^1,12–17^, with much of this work performed at the chromosome scale. In the first released genome assembly of *O. granulata*, the assembled genome sequences were not anchored to the chromosomes^11^. This undoubtedly limits the use of *O. granulata* as a basic *Oryza* lineage to accurately infer the genome evolution of *O. granulata* compared to other rice species at the chromosome level.

*O. granulata* is naturally distributed in South Asia, including China, India, Cambodia, Indonesia, Laos, Myanmar, Nepal, the Philippines, Sri Lanka, and Thailand^18^. It is seriously threatened due to ongoing human disturbance and rapid deforestation^19^. Previous population genetic studies revealed that this species possesses fairly low levels of genetic diversity within populations but high genetic differentiation among populations^20,21^. The considerable genomic diversity detected through pan-genome analysis demonstrates that *de novo* assembly of more than one genome helps reveal the origin and evolutionary forces of population structure and levels of genomic diversity^22^. Thus, sequencing an additional genome of *O. granulata* from a genetically different population compared with the previously sequenced accession collected in India^11^ is needed.

The availability of a chromosome-scale genome of *O. granulata* will lay the foundation for further evolutionary studies as well as the improvement of desired agronomic traits relevant to rice breeding programmes. Here, we present a new chromosome-scale genome of *O. granulata* assembled *de novo* using the Illumina and Hi-C sequencing platforms. In contrast to the previously sequenced *O. granulata* accession (IRGC Acc. No. 102117) from India, the sequenced plant was collected in Yunnan, China, and thus, the plants were geographically separated. The obtained genome assembly will provide novel insights into the genomic diversity and genome evolution of the genus *Oryza* and enhance the exploration of precious wild rice germplasm resources.

## Methods

### Plant material collection, total DNA isolation and genome sequencing

For genome sequencing, we collected dozens of *O. granulata* plants from Menghai County, Yunnan Province, China, which were planted in the greenhouse of the Kunming Institute of Botany, Chinese Academy of Sciences. Fresh and healthy leaves were harvested from the best-growing individual and immediately frozen in liquid nitrogen, followed by preservation at −80 °C in the laboratory prior to DNA extraction. High-quality genomic DNA was extracted from leaves using a modified CTAB method^23^. RNase A was used to remove RNA contaminants. The quality and quantity of the extracted DNA were examined using a NanoDrop 2000 spectrophotometer (NanoDrop Technologies, Wilmington, DE, USA) and electrophoresis on a 0.8% agarose gel, respectively.

A total of three 260-bp short-insert libraries and five long-insert libraries (3 kb, 10 kb and 20 kb) were prepared following Illumina’s instructions. Then, the Illumina HiSeq. 2000 (PE100 and PE101) and HiSeq. 2500 (PE125 and PE150) platforms were employed for whole-genome sequencing according to the standard Illumina protocols (Illumina, San Diego, CA, USA). In total, we generated approximately 133.38 Gb (~168.41×) of raw data (Table 1).

**Table 1:** Libraries and read statistics used for the *O. granulata* genome assembly.

Libraries	Illumina sequencer	Insert size (bp)	Read length (bp)	Raw data (Mb)	Raw sequence coverage (×)
Paired-End	HiSeq. 2500	260	150	18,198.87	22.98
	HiSeq. 2500	260	150	19,450.86	24.56
	HiSeq. 2500	260	150	18,386.95	23.22
Mate Pairs	HiSeq. 2000	2,940	100	18,499.36	23.36
	HiSeq. 2000	2,920	100	16,957.87	21.41
	HiSeq. 2000	2,980	100	15,201.03	19.19
	HiSeq. 2500	8,960	125	15,150.44	19.13
	HiSeq. 2000	19,800	101	11,536.84	14.57
Hi-C	HiSeq. X Ten	200–500	150	109,413.57	138.15

Note that the sequencing coverage is calculated by the genome size of 792 Mb.

### Hi-C data preparation

We constructed Hi-C libraries using young leaves collected from the same individual plant of *O. granulata* for high-quality DNA isolation by following the standard protocol described previously with certain modifications^24^. Approximately 5-g leaf samples were cut into minute pieces and cross-linked by 2% formaldehyde solution at room temperature for 15 minutes. Then, the sample was mixed with excess 2.5 M glycine to stop the cross-linking reaction and neutralize the remaining formaldehyde. The Hi-C library was constructed and sequenced by Annoroad Genomics (Beijing, China) with the standard procedure described as follows. The cross-linked DNA was extracted and then digested with *Mbo*I restriction enzyme. The sticky ends of the digested fragments were biotinylated and proximity ligated to form ligation junctions that were enriched for and then ultrasonically sheared to a size of 200–500 bp. The biotin-labelled DNA fragments were pulled down and ligated with Illumina paired-end adapters and then amplified by PCR to produce the Hi-C sequencing library. The library was sequenced with the Illumina HiSeq. X Ten (PE150) platform, and a total of ~109.41 Gb (~138.15×) of raw sequencing data was produced (Table 1).

### RNA isolation and transcriptome sequencing

A total of seven tissues representing different developmental stages of *O. granulata* were sampled to generate the RNA-Seq data needed for subsequent genome annotation. These tissues included panicles at three different stages of flower development, flag leaves, and stems and the shoots and roots of three-leaf seedlings. Because of the low germination rate of *O. granulata*, seedlings were germinated from seeds harvested from multiple plants, while the remaining tissues were sampled from the individual used for genome sequencing. All collected samples were quickly frozen in liquid nitrogen and stored in a refrigerator at −80 °C before RNA extraction.

RNA was individually extracted from each tissue using TRI reagent (Molecular Research Centre, Inc., Cincinnati, OH, USA), according to the instructions provided with the reagents. Seven libraries were constructed and sequenced by Biomarker Technologies (Beijing, China) on the Illumina HiSeq. 2500 platform with a read length of 126 bp. In total, ~21.8 Gb of high-quality data was obtained and used for subsequent assembly after filtering the low-quality and duplicated reads caused by PCR amplification (Table 2).

**Table 2:** Clean RNA-Seq data of *O. granulata* from seven tissues.

RNA source tissues	Read length (bp)	Number of paired-end reads	Clean data (bp)
Panicles at the booting stage	126	15,198,948	3,360,266,967
Panicles when flowering	126	14,134,157	3,026,281,452
Panicles at the grain-filling stage	126	14,272,224	3,105,851,172
Flag leaves	126	15,492,612	3,382,030,990
Stem	126	14,072,253	3,059,093,232
Shoots of seedlings	126	13,954,519	3,029,673,209
Roots of seedlings	126	13,081,148	2,845,575,334
Total		100,205,861	21,808,772,356

### Estimation of genome size

The genome size of *O. granulata* was estimated using two methods, including *k*-mer frequency distribution and flow cytometric analysis. We first estimated and validated the genome size of *O. granulata* using flow cytometric analysis. A total of 40-50 mg of fresh leaves was collected for sample preparation using the OTTO method^25,26^. Nuclear samples were analysed using a BD FACSCalibur (BD Biosciences, USA) flow cytometer. CellQuest software (BD Biosciences, USA) was used to analyse the flow cytometry results and gate all cells of interest. Here, CV = D/M × 100%, where D is the standard deviation of the cell distribution and M is the average of the cell distribution. The average coefficient of variation (CV) was used to evaluate the results, with CV < 5% considered reliable. Nuclear DNA content was calculated as a linear relationship between the ratios of 2C-value peaks of the sample and standard.

**Fig. 1:**
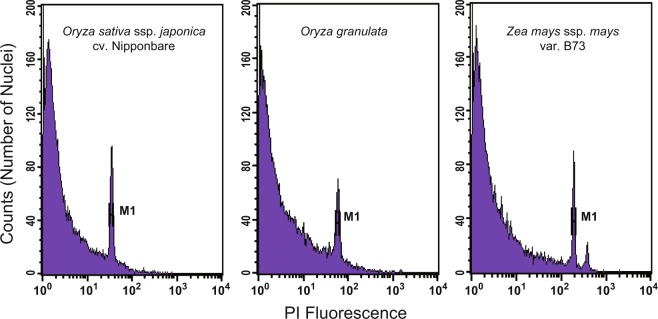
Cytogram of the fluorescence intensity of *O. sativa* ssp. *japonica* cv. Nipponbare, *O. granulata* and *Z. mays* ssp. *mays* var. B73 nuclei isolated with Otto’s buffer. All CV values were <5%.

**Fig. 2:**
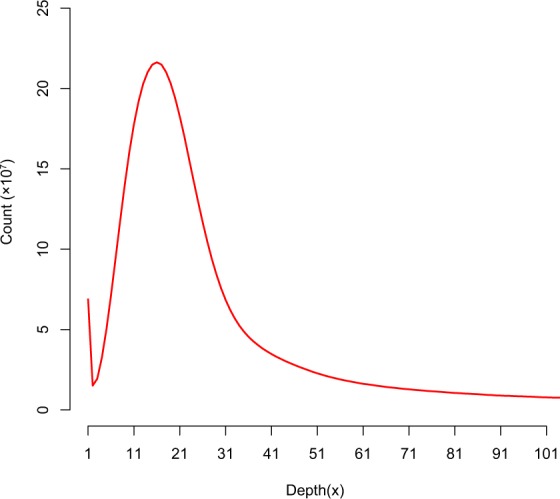
The 17-mer distribution of sequencing reads from *O. granulata*. The occurrence of 17-mers was calculated using GCE based on the sequencing data from short-insert-size libraries (insert size ≤500 bp) of *O. granulata*.

When *O. sativa* ssp. *japonica* cv. Nipponbare (~389 Mb)^9,12^ and *Zea mays* ssp. *mays* var. B73 (2,300 Mb)^27^ were employed as inner standards, the estimated genome size of *O. granulata* was ~672 Mb and ~707 Mb, respectively, both of which were smaller than the previous estimate (882 Mb) (Fig. 1). Meanwhile, we generated the 17-mer occurrence distribution of sequencing reads from short libraries using the *k*-mer method (Fig. 2). Then, we estimated the genome size to be ~792 Mb, and the proportion of repeat sequences and heterozygosity rate of the genome were determined to be approximately 70.7% and 0.76%, respectively, using GCE^28^.

### Genome assembly

We assembled the *O. granulata* genome using ALLPATHS-LG^29^ and SSPACE^30^. First, the high-quality paired-end Illumina reads from short-insert-size libraries were assembled into contig sequences using ALLPATHS-LG. This process yielded assembly results with a contig N50 value of 22,359 bp and total length of ~732.33 Mb. Second, all mate-pair reads with large insert sizes (≥2 kb) were aligned onto the preassembled contigs. According to the order and distance information, the assembled contigs were further elongated and eventually combined into scaffolds using SSPACE. We closed the gaps that might be repeat sequences masked during the construction of scaffolds using GapCloser^31^. Briefly, all paired-end sequencing reads were first mapped onto the assembled scaffolds, and then those read pairs with one read well aligned to the contigs and another located in a gap region were retrieved and locally assembled to close gaps. Consequently, the *O. granulata* genome assembly had a total length of ~736.66 Mb, which accounted for ~93% of the genome size estimated by *k*-mer analysis, containing 2,393 scaffolds (N50 = 916.3 kb; N90 = 239.8 kb) and 29,963 contigs (N50 = 43.9 kb; N90 = 12.0 kb). There were 1,146 scaffolds with lengths >100 kb, among which the largest scaffold had a sequence length of 4,040,447 bp (Table 3).

**Table 3:** Scaffold length distribution of the *O. granulata* genome.

Scaffold length	Number	Scaffold length (bp)	Average length (bp)	Percentage (%)
>1 kb	2,389	736,656,379	308,353	99.99
>10 kb	1,582	734,295,743	464,156	99.68
>50 kb	1,321	727,772,083	550,925	98.79
>100 kb	1,146	715,037,410	623,941	97.06
>200 kb	925	682,041,900	737,342	92.59
>300 kb	738	635,988,431	861,772	86.33
>500 kb	518	550,118,292	1,062,004	74.68
>800 kb	290	403,838,490	1,392,546	54.82
>1 Mb	215	336,057,751	1,563,059	45.62

Three approaches were used to evaluate the completeness and accuracy of this genome assembly. First, we mapped all high-quality reads (~186.8 million, ~87×) from short-insert-size libraries back to the assembly using BWA (Burrows-Wheeler Aligner)^32^, showing good alignments with an average mapping rate of ~99.46%. Second, the completeness of genome assembly and gene prediction was assessed with BUSCO (Benchmarking Universal Single-Copy Orthologs)^33^ according to collections from the Embryophyta lineage. Our gene predictions revealed 1,390 (96.53%) of the 1,440 highly conserved core proteins in the Embryophyta lineage. Third, the RNA sequencing reads generated in this study were assembled into a total of 137,380 transcripts using Trinity^34^, which had an N50 value of 1,035 bp and a total length of ~88.8 Mb. Then, they were aligned back to the genome assembly using GMAP^35^. Our results showed that a total of 89,977 transcripts could be successfully aligned to the genome assembly with a mapping rate of 65.5%. After filtering the low-quality reads using Trimmomatic^36^, clean paired-end reads of Hi-C data were mapped to the assembled scaffolds by BWA-MEM^32^. Finally, 1,265 (723.2 Mb, 98.2% of the assembled length) of 2,393 scaffolds were mapped, grouped and ordered into 12 chromosomes using LACHESIS^37^ (Table 4).

**Table 4:** Assembly statistics of the *O. granulata* genome sequence.

Chromosome ID	Scaffold number	Chromosome length (bp)
1	134	80,745,213
2	137	77,995,952
3	154	77,713,834
4	108	71,071,801
5	95	64,488,131
6	115	58,352,620
7	111	57,695,860
8	102	55,414,587
9	79	54,629,218
10	66	45,527,259
11	82	44,217,632
12	82	35,346,218
Unmapped	1,128	13,587,283
Total	2,393	736,785,608

### Annotation of protein-coding genes

We predicted protein-coding genes of the *O. granulata* genome using three methods, including *ab initio* gene prediction, homology-based gene prediction and RNA-Seq-aided gene prediction. Prior to gene prediction, the assembled *O. granulata* genome was hard and soft masked using RepeatMasker^38^. We adopted Augustus^39–41^ and SNAP^42^ to perform *ab initio* gene prediction. Models used for each gene predictor were trained from a set of high-quality proteins generated from the RNA-Seq dataset. We used Exonerate^43^ and GeneWise^44,45^ to conduct homology-based gene prediction. First, the protein sequences were aligned to the *O. granulata* genome assembly using Exonerate with the default parameters. Second, given that GeneWise is a time-consuming program to run, we mapped the protein sequences from *O. sativa* ssp. *japonica* cv. Nipponbare (MSU 7.0) to the *O. granulata* genome using GenBlastA^46^ prior to GeneWise prediction. Homologous genomic fragments of the target genes together with their 5-kb upstream and downstream flanking sequences were then extracted using an in-house Perl script. Finally, GeneWise was used to align them against the corresponding proteins to determine gene structures. To carry out RNA-Seq-aided gene prediction, we first assembled clean RNA-Seq reads into transcripts using Trinity^34^, which were then aligned to our genome assembly using PASA^47^. The output included a set of consistent and non-overlapping sequence assemblies, which were used to describe the gene structures.

We combined all gene structures obtained from the three above-mentioned sets of predictions, including *ab initio* gene predictions and protein and transcript alignments, with the weighted consensus gene set using EVidenceModeler (EVM)^48^. To perform further filtering, the genes with peptide lengths shorter than 50 amino acids and/or containing inner stop codons were removed. In total, 40,131 protein-coding genes with an average length of 3,152 bp were predicted in the assembled *O. granulata* genome.

**Fig. 3:**
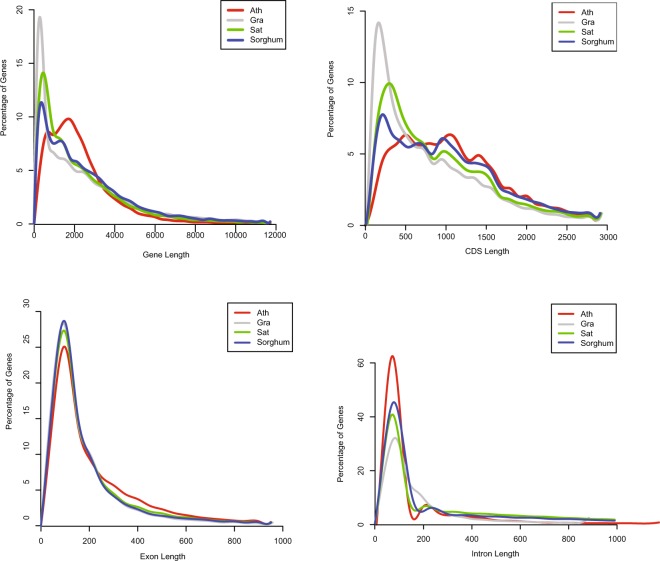
Comparisons of gene features among *O. granulata* and the three other species (*A. thaliana*, *S. bicolor* and *O. sativa*). Gene features include gene length, CDS length, exon length and intron length.

To assess the quality of gene prediction, we compared the length distributions of protein-coding genes, coding sequences (CDS), exons and introns with those from the other four species (*Arabidopsis thaliana, Sorghum bicolor, Z. mays* and *O. sativa*), among which we did not observe any obvious differences in the length distribution of gene features (Fig. 3; Table 5). Then, we surveyed the proportion of our predicted *O. granulata* gene sets supported by RNA-Seq and homologous proteins. We aligned the assembled transcripts against our gene predictions using the BLAST program^49,50^. Only hits with a coverage ≥80% and an identity ≥90% were retained. Our analysis showed that approximately 47.58% (19,094) of the predicted gene models were supported by RNA-Seq data. Next, we downloaded protein sequences of *O. sativa* ssp. *japonica* cv. Nipponbare and aligned them to the predicted gene models using BLAST. We filtered those hits with an identity <30% or a gene coverage <80%. We found that 23,871 gene models, accounting for approximately 59.48% of the total genes, were supported by evidence of homologous proteins in rice. Combining genes validated by the two above-described methods, 28,823 genes, representing ~71.82% of the total *O. granulata* gene set, were supported by RNA-Seq and/or homologous proteins (Table 6).

**Table 5:** Comparison of gene models among *O. granulata* and *A. thaliana* from Capparidales and the three grasses, namely, rice, maize and sorghum.

	*O. granulata*	*O. sativa*	*S. bicolor*	*Z. mays*	*A. thaliana*
Genome size (Mb)	737	373	727	2,068	120
Gene number (#)	40,131	39,045	32,824	40,602	27,416
Gene models (#)	49,486	49,066	39,195	40,602	35,386
Gene length (Mb)	125.53	111.45	120.86	170.84	60.48
Coding sequences (Mb)	35.78	41.56	38.60	44.70	33.40
Number of introns (#)	125,141	130,147	115,947	166,986	123,389
Total intron length (Mb)	78.84	54.72	51.76	105.44	11.62
Avg intron length (bp)	630	420	446	631	94

**Table 6:** Validation and functional annotation of the *O. granulata* protein-coding genes.

	Methods	Number	Percentage (%)
Validation	Protein supported	23,871	59.48
	RNA-Seq supported	19,094	47.58
	Protein or RNA-Seq supported	28,823	71.82
Functional annotation	Swiss-Prot	22,348	55.69
	KEGG	7,365	18.35
	GO	19,458	48.49
	PFAM	21,533	53.66
	NR	27,522	68.58
	Total annotated	34,436	85.81

Gene functions were inferred according to the best match of the alignments to the National Center for Biotechnology Information (NCBI) Non-Redundant (NR) and Swiss-Prot protein databases using BLASTP^49,50^ and the Kyoto Encyclopedia of Genes and Genomes (KEGG) database with an E-value threshold of 1E-5. The motifs and domains within gene models were identified by PFAM databases^51^. Gene Ontology (GO) IDs for each gene were obtained from Blast2GO^52^. In total, approximately 85.81% of the predicted protein-coding genes of *O. granulata* could be functionally annotated with known genes, conserved domains, and Gene Ontology terms (Table 6).

### Annotation of non-coding RNA genes

Five different types of non-coding RNA genes, namely, transfer RNA (tRNA) genes, ribosomal RNA (rRNA) genes, small nucleolar RNA (snoRNA) genes, small nuclear RNA (snRNA) genes and microRNA (miRNA) genes, were predicted using *de novo* and homology search methods. We used tRNAscan-SE algorithms^53^ with default parameters to identify the genes associated with tRNA, which is an adaptor molecule composed of RNA used in biology to bridge the three-letter genetic code in messenger RNA (mRNA) with the twenty-letter code of amino acids in proteins. The rRNA genes (8S, 18S, and 28S), which are the RNA components of the ribosome and associated with the enzyme representing the site of protein synthesis in all living cells, were predicted using RNAmmer algorithms^54^ with default parameters. snoRNAs are a class of small RNA molecules that guide chemical modifications of other RNAs, mainly ribosomal RNAs, transfer RNAs and small nuclear RNAs. The snoRNA genes were annotated using Snoscan^55^ with the yeast rRNA methylation sites and yeast rRNA sequences provided by the Snowscan distribution. snRNA is a class of small RNA molecules that are found within the nucleus of eukaryotic cells. They are involved in a variety of important processes, such as RNA splicing (removal of introns from hnRNA), regulation of transcription factors (7SK RNA) or RNA polymerase II (B2 RNA), and maintenance of telomeres. The snRNA genes were identified by Infernal software against the Rfam database with default parameters^56,57^.

The miRNA genes were annotated in two steps. First, we downloaded the existing rice miRNA entries from miRBase^58^. Then, the conserved miRNAs were identified by mapping all miRBase-recorded rice miRNA precursor sequences against the assembled *O. granulata* genome using BLASTN with cut-offs at an identity >60% and a query coverage >60%. Second, when a miRNA was mapped to the target *O. granulata* genome, the surrounding sequence was checked for hairpin structures. The loci with miRNA precursor secondary structures were annotated as miRNA genes.

We annotated a total of 1,003 tRNA genes, 221 rRNA genes, 295 snoRNA genes, 101 snRNA genes and 257 miRNA genes belonging to 50 miRNA families in the *O. granulata* genome (Table 7). To investigate miRNA-target genes involved in important biological pathways, the target genes of miRNAs were predicted using the psRNATarget server with default parameters. Finally, 963 miRNA-target sites were identified. The protein sequences of these target genes were blasted against *O. sativa* proteins in the Rice Genome Annotation Project Database^59^ using the BLASTP program. The results were imported into the agriGO^60^ server by comparing them with the whole set of protein-coding genes of *O. sativa* as a background. KO (KEGG Orthology) annotation of target genes was implemented using the BlastKOALA program^61^ with the eukaryote gene database.

**Table 7:** Conserved non-coding RNA genes in the *O. granulata* genome.

ncRNA types	Number	Average length (bp)	Total length (bp)	% of genome
tRNA	1,003	74.9	75,160	0.0103
rRNA (8S)	181	113.5	20,548	0.0028
rRNA (18S)	21	1,642.3	34,487	0.0047
rRNA (28S)	19	4,139.9	78,659	0.0108
snoRNA	295	113.6	33,501	0.0046
snRNA	101	142.7	14,408	0.0020
miRNA	257	114.7	29,471	0.0040

### Annotation of repeat sequences

We identified the known TEs within the *O. granulata* genome using RepeatMasker with the Repbase TE library^62,63^. RepeatProteinMask searches were also conducted using the TE protein database as a query library. The annotation of repeat sequences of the *O. granulata* genome is summarized in Table 8. The annotation showed that approximately 61.98% (456.6 Mb) of the assembled genome consisted of repeat sequences, and the proportion of repeat sequences varied largely from one type to another. DNA transposons and other repeats contributed only ~9.83% and ~1.1% to the assembled genome, respectively. In contrast, retrotransposons represented half (~51.05%) of the genome assembly.

**Table 8:** Summary of the annotated repeat sequences in the *O. granulata* genome.

Transposable elements	Length (bp)	Percentage (%)
DNA transposons	72,407,795	9.83
*En-Spm*	10,454,636	1.42
*Harbinger*	10,685,997	1.45
Maverick	900,730	0.12
*MuDR*	31,123,966	4.23
TcMar-Stowaway	7,334,046	1.00
Tourist	62,565	0.01
*hAT*	8,611,368	1.17
Helitron	893,911	0.12
Others	2,340,576	0.32
RNA transposons	376,029,522	51.05
Non-LTR retrotransposons	1,584,873	0.22
LINE	1,459,061	0.20
SINE	125,812	0.02
LTR retrotransposons	374,444,649	50.83
*Copia*	41,126,935	5.58
*Gypsy*	278,699,663	37.83
Others	54,618,051	7.41
Other repeats	8,130,028	1.10
Low complexity	764,382	0.10
Simple repeats	3,685,309	0.50
Unknown	3,680,337	0.50
Total	456,567,345	61.98

We constructed a *de novo* repeat library of the *O. granulata* genome using RepeatModeler, which can automatically execute two core *de novo* repeat-finding programs, namely, RECON^64^ and RepeatScout^65^, to comprehensively conduct, refine and classify consensus models of putative interspersed repeats for the *O. granulata* genome. Furthermore, we performed a *de novo* search for long terminal repeat (LTR) retrotransposons against the *O. granulata* genome sequences using LTR_STRUC^66^. All intact LTR retrotransposons were classified into Ty1/*copia*, Ty3*/gypsy* and unclassified groups according to both reverse transcriptase (RT) sequence similarity and the order of ORFs using Pfam^51^. The RT sequences were retrieved from each retrotransposon element and further checked by homology searches using ClustalW^67^ against the published RTs that were downloaded from the *Gypsy* Database (GyDB)^68^. LTR retrotransposons (~50.83%) represented most of the RNA transposons in the *O. granulata* genome, accounting for approximately 43.41% of the assembly. They belonged to two types of LTR retrotransposon superfamilies: Ty1/*copia* and Ty3/*gypsy* (~5.58% and ~37.83%, respectively) (Table 8).

We also identified tandem repeats using the Tandem Repeat Finder (TRF) package^69^ and the non-interspersed repeat sequences, including low-complexity repeats, satellites and simple repeats, using RepeatMasker (Table 8). A total of six types of simple sequence repeats (SSRs), from mono- to hexa-nucleotides, were identified using the MISA (MIcroSAtellite) identification tool^70^. The minimum repeat unit size was set at twelve for mono-nucleotides, at six for di-nucleotides, at four for tri-nucleotides, and at three for tetra- to hexa-nucleotides. As a result, a total of 183,339 SSRs were detected in the *O. granulata* genome. Of these, tri-nucleotide SSRs accounted for the largest proportion, both in quantity and sequence length, followed by tetra-nucleotides, di-nucleotides and other types (Table 9). These SSRs will provide valuable molecular markers to assist rice breeding programmes.

**Table 9:** Occurrence of simple sequence repeats (SSRs) in the *O. granulata* genome.

Repeat type	Number	Average length (bp)	Total length (kb)	Proportion (%)
Mono-nucleotide	9,397	14	132.89	5.14
Di-nucleotide	35,467	16	576.31	22.28
Tri-nucleotide	64,905	13	860.99	33.28
Tetra-nucleotide	53,484	13	676.26	26.14
Penta-nucleotide	11,720	16	182.83	7.07
Hexa-nucleotide	8,366	19	157.78	6.10
Total	183,339	14	2,587.06	100

Note that the minimum repeat unit size was set at twelve for mono-nucleotides, at six for di-nucleotides, at four for tri-nucleotides, and at three for tetra- to hexa-nucleotides.

## Data Records

All sequencing reads have been deposited into the NCBI Sequence Read Archive (SRA)^71^ and BIG Genome Sequence Archive^72^. The assembled genome sequence is available from the NCBI^73,74^ and BIG Genome Warehouse^75^. The protein-coding gene, non-coding gene, and repeat sequence annotation results and functional prediction results are available from the Figshare database^76^.

## Technical Validation

### RNA integrity

Before constructing RNA-Seq libraries, the concentration and amount of total RNA were separately evaluated using a NanoDrop 2000 UV-VIS spectrophotometer (NanoDrop Technologies, Wilmington, DE, USA), and the rRNA ratio and RNA integrity were estimated using an Agilent 2100 Bioanalyzer (Agilent Technologies, Palo Alto, CA, USA). For each tissue, only total RNAs with a total amount ≥15 μg, a concentration ≥400 ng/μl, an RNA integrity number (RIN) ≥7, and an rRNA ratio ≥1.4 were used to construct a cDNA library according to the manufacturer’s instructions (Illumina, USA).

### Quality filtering of Illumina sequencing raw reads

To eliminate adapter contaminants and potential sequencing errors, using Trimmomatic^36^, we removed the following five types of reads: (1) reads with ≥10 bp derived from the adapter sequences (allowing ≤10% mismatches); (2) reads with unidentified bases (Ns) constituting ≥10% of their length; (3) reads with ≥40% low-quality bases (Phred score ≤5); (4) reads caused by PCR duplications (i.e., read 1 and read 2 of two paired-end reads that were completely identical); and 5) reads with a *k*-mer frequency ≤3 (aiming to minimize the influences of sequencing errors). These five filtering processes resulted in a total of ~108.72 Gb (~137.27×) of high-quality data, which were retained and used for subsequent analysis (Table 1).

### Comparisons of the genome assemblies and annotation

We produced high-depth sequencing data for *O. granulata* using the Illumina and Hi-C sequencing platforms. Then, we *de novo* assembled an ~736.66 Mb genome assembly of *O. granulata* comprising 2,393 scaffolds with a scaffold N50 of ~916.3 kb (Online-only Table 1). The contig N50 was ~43.9 kb, which was higher than that obtained for the genome assemblies of other *Oryza* species with similar second-generation sequencing technology^17^. With Hi-C data, for the first time, we anchored approximately 98.2% of the genome assembly into the twelve pseudo-chromosomes. Due to the short reads sequenced by the Illumina platform and a large number of repeat sequences, the total lengths of genome assembly (~736.66 Mb) and repetitive sequences (~456.57 Mb) are shorter than those in the previous genome assembly (~776.96 Mb and ~528.04 Mb, respectively)^11^ (Online-only Table 1). This may be attributed to the ~21 × PacBio data overcoming the above-mentioned difficulties to some extent, resulting in the assembly of an additional portion of repetitive sequences. However, we obtained fewer scaffolds but a longer scaffold N50 compared to those in the previous genome assembly^11^. We predicted 40,131 protein-coding genes and observed that the gene and ncRNA annotations were somewhat better than those for the previous genome assembly (Online-only Table 1). This was also evidenced by the evaluation using BUSCO, showing that 1,390 genes (~96.53%) were completely identified, which is somewhat better than the number for the previous genome assembly^11^. Thus, the newly released genome assembly, which has good continuity and integrity, is comparable to other sequenced *Oryza* genomes.

## Data Availability

The sequence data were generated using the software provided by the sequencing platform manufacturer and processed with commands provided for the public software cited in the manuscript. No custom computer code was generated in this work. The following bioinformatic tools and versions were used to generate all results as described in the main text. Default parameters were used if not stated. 1. CellQuest version 5.1. 2. GCE (Genome Characteristics Estimation) version 1.0.0 was used to estimate genome size, ftp://ftp.genomics.org.cn/pub/gce/. 3. ALLPATHS-LG version 48894 was used for genome assembly, http://software.broadinstitute.org/allpaths-lg/blog/. 4. SSPACE version 3.0 was used for genome assembly scaffolding, https://www.baseclear.com/services/bioinformatics/basetools/sspace-standard/. 5. GapCloser version 1.12 was used to fill the gaps between scaffolds, http://soap.genomics.org.cn/about.html. 6. BWA (Burrows-Wheeler Aligner) version 0.7.15 was used for short read mapping, https://github.com/lh3/bwa/. 7. BUSCO (Benchmarking Universal Single-Copy Orthologs) was used to check the completeness of the genome assembly, with coverage ≥ 90% and identity ≥ 90% parameters, https://gitlab.com/ezlab/busco/. 8. Trinity version v2.0.6 was used to assemble the RNA sequencing reads, https://github.com/trinityrnaseq/trinityrnaseq. 9. GMAP version 2014-10-2 was used to map the assembled transcripts to the genome sequence with coverage ≥ 90% and identity ≥ 90% parameters, http://research-pub.gene.com/gmap. 10 LACHESIS was used for ultra-long-range scaffolding with Hi-C data with CLUSTER_N = 12, CLUSTER_MIN_RE_SITES = 300, CLUSTER_MAX_LINK_DENSITY = 8, ORDER_MIN_N_RES_IN_TRUNK = 100, and ORDER_MIN_N_RES_IN_SHREDS = 10 parameters, http://shendurelab.github.io/LACHESIS/. 11. RepeatMasker version 4.0.3 was used to mask the repeat sequences in the genome, http://repeatmasker.org/. 12. Augustus version 2.7 was used for *de novo* gene prediction, http://augustus.gobics.de/. 13. SNAP version 2006-07-28 was used for *de novo* gene prediction, https://github.com/KorfLab/SNAP. 14. Exonerate version 2.2.0 was used to align proteins to the genome sequence, https://www.ebi.ac.uk/~guy/exonerate/. 15. GeneWise version 2-2-0 was used to predict gene structure using similar protein sequences, http://www.ebi.ac.uk/~birney/wise2. 16. GenBlastA version 1.0.1 was used to link the high-scoring pairs (HSPs), http://genome.sfu.ca/genblast/download.html. 17. PASA (Program to Assemble Spliced Alignments) was used to exploit gene structure using transcripts, http://pasapipeline.github.io/. 18. EVidenceModeler (EVM) version 1.1.1 was used to combine gene predictions generated from different methods into consensus gene structures, http://evidencemodeler.github.io/. 19. BLAST version 2.2.28 was used to find regions of local similarity between sequences, https://blast.ncbi.nlm.nih.gov/Blast.cgi/. 20. KEGG (Kyoto Encyclopedia of Genes and Genomes), https://www.kegg.jp/. 21. Pfam database: http://pfam.xfam.org/. 22. Blast2GO: https://www.blast2go.com/. 23. The tRNAscan-SE algorithm (version 1.23) was used for the identification of tRNA genes, http://lowelab.ucsc.edu/tRNAscan-SE. 24. The RNAmmer algorithm was used for the identification of rRNA genes, http://www.cbs.dtu.dk/services/RNAmmer/. 25. Snoscan version 1.0 was used for the identification of snoRNA genes, http://lowelab.ucsc.edu/snoscan/. 26. INFERNAL version 1.1.2 was used for the identification of snRNA genes, http://eddylab.org/infernal/. 27. Rfam database release 9.1, rfam.xfam.org/. 28. miRBase release 21, www.mirbase.org/. 29. psRNATarget server; parameters: maximum expectation = 3.0, length for complementary scoring = 20 bp, target accessibility – allowed maximum energy to unpair the target site = 25.0, flanking length around the target site for target accessibility analysis: 17 bp upstream and 13 bp downstream, and range of central mismatch leading to translational inhibition = 9~11 bp, http://plantgrn.noble.org/psRNATarget/. 30 Rice Genome Annotation Project Database, http://rice.plantbiology.msu.edu/. 31. agriGO server, http://bioinfor.cau.edu.cn/agiGO/. 32. BlastKOALA, http://kegg.jp/blastkoala/. 33. Repbase TE library (version released on January 31, 2014). 34. RepeatProteinMask, http://www.repeatmasker.org/RepeatProteinMask.html. 35. RepeatModeler version 1.0.10 was used for *de novo* repeat family identification and modelling, http://www.repeatmasker.org/RepeatModeler/. 36. RECON version 1.08. 37. RepeatScout version 1.0.5. 38. LTR_STRUC was used for the identification of LTR retrotransposons, http://www.mcdonaldlab.biology.gatech.edu/ltr_struc.htm. 39. ClustalW was used to perform multiple sequence alignment, https://www.genome.jp/tools-bin/clustalw. 40. *Gypsy* Database (GyDB), http://gydb.org/. 41. Tandem Repeat Finder (TRF) version 4.07b was used to find the tandem repeats in the genome with the parameters Match = 2, Mismatch = 7, Delta = 7, PM = 80, PI = 10, Minscore = 50, and MaxPeriod = 12, https://tandem.bu.edu/trf/trf.html. 42. RepeatMasker version 4.0.3 was used to mask the repeat sequences in the genome with the parameter -noint, http://www.repeatmasker.org. 43. The MISA (MIcroSAtellite) identification tool was used for the identification and localization of microsatellites, http://pgrc.ipk-gatersleben.de/misa/. 44. Trimmomatic version 0.33 was used for the quality filtering of sequencing reads, http://www.usadellab.org/cms/index.php?page=trimmomatic.

## Online-only Table

**Online-only Table 1:** Comparison of the two *O. granulata* genome assemblies.

	Entries	This study	IRGC Acc. No. 102117^11^
Genome sequencing	Source country	China	India
	Genome size (Mb) *	792	785
	Sequencing technology	Illumina; Hi-C	Illumina; PacBio
	Raw Illumina data (Gb)	133.38	105.157
	Sequence coverage (×)**	167	131
	Raw Hi-C/PacBio data (Gb)	109.41	16.615
	Sequence coverage (×)**	137	21
Assembly statistics	Assembly size (Mb)	736.66	776.96
	Whole-genome coverage (%)	93	98.1
	Contig N50 (kb)	43.9	262.05
	Contig number (#)	29,963	4,618
	Scaffold N50 (kb)	916.3	262.05
	Scaffold number (#)	2,393	4,618
	Largest scaffold (Mb)	4.04	1.59
	Length of anchored scaffolds (Mb)	723.2	—
	Anchoring rate (%)	98.2	—
	GC content (%)	45.87	46.32
Gene annotation	Gene number (#)	40,131	40,116
	Functionally annotated gene number (#)	34,436	33,901
	Complete BUSCO (%)	96.53	95
	Total gene length (Mb)	125.53	102.74
	Average gene length (bp)	3,152	2,561.19
	Total CDS length (bp)	35.78	40.61
	Average CDS length (bp)	892	1,012.28
	Number of exons (#)	165,272	162,369
	Average exon length (bp)	283	250.1
	Average exons per gene	4.1	4.05
	Total intron length (Mb)	78.84	62.14
	Number of introns (#)	125,141	122,253
	Average intron length (bp)	630	508
ncRNA annotation	tRNA length (bp)	75,160	82,079
	rRNA length (bp)	133,694	99,297
	miRNA length (bp)	29,471	30,787
Repeat sequence annotation	Total repeat length (Mb)	456.567	528.04
	Repeat percentage (%)	61.98	67.96
	DNA transposon length (bp)	72,407,795	68,393,246
	LINE length (bp)	1,459,061	7,169,231
	SINE length (bp)	125,812	59,741
	LTR length (bp)	374,444,649	460,976,797
	*Copia* (bp)	41,126,935	54,901,814
	*Gypsy* (bp)	278,699,663	407,036,517
	Others (bp)	54,618,051	28,158,459
	Other length (bp)	8,130,028	1,696,213

*The genome size was estimated by the *k*-mer method;

**The genome size was estimated to be 800 Mb.

## References

[1] Wang M (2014). The genome sequence of African rice (*Oryza glaberrima*) and evidence for independent domestication. Nat. Genet..

[2] Guo YL, Ge S (2006). Advances in the study of systematics and evolution of the tribe Oryzeae (Poaceae). Acta Phytotaxon. Sin..

[3] Heer, O. *Flora Tertiaria Helvetiae - Die tertiäre Flora der Schweiz*. (J. Würster & Compagnie, 1855).

[4] Tang L (2010). Phylogeny and biogeography of the rice tribe (Oryzeae): evidence from combined analysis of 20 chloroplast fragments. Mol. Phylogenet. Evol..

[5] Department of Agronomy, Kwangtung Agrieultural and Forestry College (1975). The species of wild rice and their geographical distribution in China. J. Genet. Genomics.

[6] The Cooperative Team of Wild Rice Resources Survey and Exploration of China (1984). A general survey and exploration of wild rice germplasm resources in China. Sci. Agric. Sinica.

[7] Fan SG, Zhang ZJ, Liu L, Liu HX, Liang CY (2000). The species, geographical distribution of wild rice and their characteristics in China. J. Wuhan Bot. Res..

[8] Ammiraju JSS (2006). The *Oryza* bacterial artificial chromosome library resource: construction and analysis of 12 deep-coverage large-insert BAC libraries that represent the 10 genome types of the genus *Oryza*. Genome Res..

[9] Goff SA (2002). A draft sequence of the rice genome (*Oryza sativa* L. ssp. *japonica*). Science.

[10] Piegu B (2006). Doubling genome size without polyploidization: Dynamics of retrotransposition-driven genomic expansions in *Oryza australiensis*, a wild relative of rice. Genome Res..

[11] Wu ZG (2018). *De novo* genome assembly of *Oryza granulata* reveals rapid genome expansion and adaptive evolution. Commun. Biol..

[12] International Rice Genome Sequencing Project (2005). The map-based sequence of the rice genome. Nature.

[13] Chen J (2013). Whole-genome sequencing of *Oryza brachyantha* reveals mechanisms underlying *Oryza* genome evolution. Nat. Commun..

[14] Li, W. *et al*. Improved hybrid *de novo* genome assembly and annotation of African wild rice, *Oryza longistaminata*, from Illumina and PacBio sequencing reads. *Plant Genome-US*, e20001 (2020). 10.1002/tpg2.20001 PMC12807249 33016624

[15] Li, W. *et al*. SMRT sequencing of the *Oryza rufipogon* genome reveals the genomic basis of rice adaptation. *Commun. Biol.* **3**, 167 (2020). 10.1038/s42003-020-0890-8 PMC7138787 32265482

[16] Stein JC (2018). Genomes of 13 domesticated and wild rice relatives highlight genetic conservation, turnover and innovation across the genus *Oryza*. Nat. Genet..

[17] Zhang Q-J (2014). Rapid diversification of five *Oryza* AA genomes associated with rice adaptation. P. Natl. Acad. Sci. USA.

[18] Vaughan, D. A. *The Wild Relatives of Rice: A Genetic Resources Handbook*. (IRRI, 1994).

[19] Gao LZ, Zhang SZ, Zhou Y, Ge S, Hong DY (1996). A survey of the current status of wild rice in China. Biodiv. Sci.

[20] Gao LZ, Ge S, Hong DY (2000). Low levels of genetic diversity within populations and high differentiation among populations of a wild rice, *Oryza granulata* Nees et. Arn. ex. Watt. from China. Int. J. Plant Sci..

[21] Gao, L. Z. *et al*. Studies on population genetic structure of *Oryza granulata* Nees et. Arn. ex. Watt. from Yunnan and its *in situ* conservation significance. *Sci. China Ser. C*, 297–302 (1999). 10.1007/BF02881755 18726505

[22] Zhao Q (2018). Pan-genome analysis highlights the extent of genomic variation in cultivated and wild rice. Nat. Genet..

[23] Porebski S, Bailey LG, Baum BR (1997). Modification of a CTAB DNA extraction protocol for plants containing high polysaccharide and polyphenol components. Plant Mol. Biol. Rep..

[24] Belton JM (2012). Hi-C: a comprehensive technique to capture the conformation of genomes. Methods.

[25] Loureiro J, Rodriguez E, Dolezel J, Santos C (2006). Comparison of four nuclear isolation buffers for plant DNA flow cytometry. Ann. Bot.-London.

[26] Huang H, Tong Y, Zhang QJ, Gao LZ (2013). Genome size variation among and within *Camellia* species by using flow cytometric analysis. Plos One.

[27] Schnable PS (2009). The B73 maize genome: complexity, diversity, and dynamics. Science.

[28] Liu, B. H. *et al*. Estimation of genomic characteristics by analyzing *k*-mer frequency in *de novo* genome projects. Preprint at, http://arxiv.org/abs/1308.2012v1 (2013).

[29] Gnerre S (2011). High-quality draft assemblies of mammalian genomes from massively parallel sequence data. P. Natl. Acad. Sci. USA.

[30] Boetzer M, Henkel CV, Jansen HJ, Butler D, Pirovano W (2011). Scaffolding pre-assembled contigs using SSPACE. Bioinformatics.

[31] Luo RB (2012). SOAPdenovo2: an empirically improved memory-efficient short-read *de novo* assembler. GigaScience.

[32] Li, H. Aligning sequence reads, clone sequences and assembly contigs with BWA-MEM. Preprint at, http://arxiv.org/abs/1303.3997v2 (2013).

[33] Simao FA, Waterhouse RM, Ioannidis P, Kriventseva EV, Zdobnov EM (2015). BUSCO: assessing genome assembly and annotation completeness with single-copy orthologs. Bioinformatics.

[34] Grabherr MG (2011). Full-length transcriptome assembly from RNA-Seq data without a reference genome. Nat. Biotechnol..

[35] Wu TD, Watanabe CK (2005). GMAP: a genomic mapping and alignment program for mRNA and EST sequences. Bioinformatics.

[36] Bolger AM, Usadel B, Lohse M (2014). Trimmomatic: a flexible trimmer for Illumina sequence data. Bioinformatics.

[37] Burton JN (2013). Chromosome-scale scaffolding of *de novo* genome assemblies based on chromatin interactions. Nat. Biotechnol..

[38] Tarailo-Graovac, M. & Chen, N. Using RepeatMasker to identify repetitive elements in genomic sequences. *Curr. Protoc. Bioinformatics* Chapter 4, Unit 4.10. (2009). 10.1002/0471250953.bi0410s25 19274634

[39] Stanke M, Steinkamp R, Waack S, Morgenstern B (2004). AUGUSTUS: a web server for gene finding in eukaryotes. Nucleic Acids Res.

[40] Stanke M, Morgenstern B (2005). AUGUSTUS: a web server for gene prediction in eukaryotes that allows user-defined constraints. Nucleic Acids Res.

[41] Stanke M (2006). AUGUSTUS: *ab initio* prediction of alternative transcripts. Nucleic Acids Res.

[42] Korf I (2004). Gene finding in novel genomes. BMC Bioinformatics.

[43] Slater GSC, Birney E (2005). Automated generation of heuristics for biological sequence comparison. BMC Bioinformatics.

[44] Birney E, Durbin R (2000). Using GeneWise in the *Drosophila* annotation experiment. Genome Res.

[45] Birney E, Clamp M, Durbin R (2004). GeneWise and genomewise. Genome Res..

[46] She R, Chu JSC, Wang K, Pei J, Chen N (2009). S. genBlastA: enabling BLAST to identify homologous gene sequences. Genome Res..

[47] Haas BJ (2003). Improving the *Arabidopsis* genome annotation using maximal transcript alignment assemblies. Nucleic Acids Res.

[48] Haas BJ (2008). Automated eukaryotic gene structure annotation using EVidenceModeler and the program to assemble spliced alignments. Genome Biol..

[49] Altschul SF (1997). Gapped BLAST and PSI-BLAST: a new generation of protein database search programs. Nucleic Acids Res.

[50] Camacho, C. *et al*. BLAST plus: architecture and applications. *BMC Bioinformatics* **10** (2009). 10.1186/1471-2105-10-421 PMC2803857 20003500

[51] Finn RD (2008). The Pfam protein families database. Nucleic Acids Res.

[52] Conesa A, Gotz S (2008). Blast2GO: a comprehensive suite for functional analysis in plant genomics. Int. J. Plant Genomics.

[53] Lowe TM, Eddy SR (1997). tRNAscan-SE: a program for improved detection of transfer RNA genes in genomic sequence. Nucleic Acids Res.

[54] Lagesen K (2007). RNAmmer: consistent and rapid annotation of ribosomal RNA genes. Nucleic Acids Res.

[55] Lowe TM, Eddy SR (1999). A computational screen for methylation guide snoRNAs in yeast. Science.

[56] Griffiths-Jones S (2005). Rfam: annotating non-coding RNAs in complete genomes. Nucleic Acids Res.

[57] Nawrocki EP, Kolbe DL, Eddy SR (2009). Infernal 1.0: inference of RNA alignments. Bioinformatics.

[58] Kozomara A, Griffiths-Jones S (2011). miRBase: integrating microRNA annotation and deep-sequencing data. Nucleic Acids Res.

[59] Kawahara Y (2013). Improvement of the *Oryza sativa* Nipponbare reference genome using next generation sequence and optical map data. Rice.

[60] Du Z, Zhou X, Ling Y, Zhang Z, Su Z (2010). agriGO: a GO analysis toolkit for the agricultural community. Nucleic Acids Res.

[61] Kanehisa M, Sato Y, Morishima K (2016). BlastKOALA and GhostKOALA: KEGG tools for functional characterization of genome and metagenome sequences. J. Mol. Biol..

[62] Jurka J (2000). Repbase Update - a database and an electronic journal of repetitive elements. Trends Genet.

[63] Jurka J (2005). Repbase update, a database of eukaryotic repetitive elements. Cytogenet. Genome Res..

[64] Bao ZR, Eddy SR (2002). Automated *de novo* identification of repeat sequence families in sequenced genomes. Genome Res.

[65] Price AL, Jones NC, Pevzner PA (2005). *De novo* identification of repeat families in large genomes. Bioinformatics.

[66] McCarthy EM, McDonald JF (2003). LTR_STRUC: a novel search and identification program for LTR retrotransposons. Bioinformatics.

[67] Larkin MA (2007). Clustal W and clustal X version 2.0. Bioinformatics.

[68] Llorens C (2011). The Gypsy Database (GyDB) of mobile genetic elements: release 2.0. Nucleic Acids Res.

[69] Benson G (1999). Tandem repeats finder: a program to analyze DNA sequences. Nucleic Acids Res.

[70] Thiel T, Michalek W, Varshney RK, Graner A (2003). Exploiting EST databases for the development and characterization of gene-derived SSR-markers in barley (*Hordeum vulgare* L.). Theor. Appl. Genet..

[71] (2019). NCBI Sequence Read Archive.

[72] (2019). BIGD Genome Sequence Archive.

[73] (2020). NCBI Assembly.

[74] Li W (2019). GenBank.

[75] (2019). BIGD Genome Warehouse.

[76] Shi C (2019). figshare.

